# Primaquine-induced haemolysis in females heterozygous for G6PD deficiency

**DOI:** 10.1186/s12936-018-2248-y

**Published:** 2018-03-02

**Authors:** Cindy S. Chu, Germana Bancone, François Nosten, Nicholas J. White, Lucio Luzzatto

**Affiliations:** 1 Shoklo Malaria Research Unit, Faculty of Tropical Medicine, Mahidol–Oxford Tropical Medicine Research Unit, Mahidol University, Mae Sot, Thailand; 2 Mahidol–Oxford Tropical Medicine Research Unit, Faculty of Tropical Medicine, Mahidol University, Bangkok, Thailand; 3 Centre for Tropical Medicine and Global Health, Nuffield Department of Medicine, University of Oxford, Oxford, UK; 4 Department of Haematology, Muhimbili University of Health and Allied Sciences, Dar-es-Salaam, Tanzania

**Keywords:** *Plasmodium vivax*, Malaria, G6PD deficiency, Primaquine, G6PD heterozygous female, Radical cure, Haemolysis, 8-aminoquinoline

## Abstract

Oxidative agents can cause acute haemolytic anaemia in persons with G6PD deficiency. Understanding the relationship between G6PD genotype and the phenotypic expression of the enzyme deficiency is necessary so that severe haemolysis can be avoided. The patterns of oxidative haemolysis have been well described in G6PD deficient hemizygous males and homozygous females; and haemolysis in the proportionally more numerous heterozygous females has been documented mainly following consumption of fava beans and more recently dapsone. It has long been known that 8-aminoquinolines, notably primaquine and tafenoquine, cause acute haemolysis in G6PD deficiency. To support wider use of primaquine in *Plasmodium vivax* elimination, more data are needed on the haemolytic consequences of 8-aminoquinolines in G6PD heterozygous females. Two recent studies (in 2017) have provided precisely such data; and the need has emerged for the development of point of care quantitative testing of G6PD activity. Another priority is exploring alternative 8-aminoquinoline dosing regimens that are practical and improve safety in G6PD deficient individuals.

## Background

Pamaquine (plasmochin, plasmoquine), the first 8-aminoquinoline to be used for the radical curative treatment of *Plasmodium vivax* malaria caused haemolysis in approximately 5–10% of the patients who received treatment with it [1]. Primaquine succeeded pamaquine as the first-line treatment for radical cure because of its greater potency and better tolerability [2]. However, primaquine still caused haemolysis in susceptible patients. The biochemical defect responsible for oxidant haemolysis was found to be red blood cell (RBC) deficiency of the enzyme glucose-6-phosphate dehydrogenase (G6PD). Initially evaluated by the US military in the Korean war, primaquine remains today the only widely available anti-malarial for the radical curative treatment of *P. vivax* malaria.

The WHO malaria treatment guidelines have long recommended the addition of primaquine to chloroquine (or now to artemisinin-based combination therapy, ACT) for the radical curative treatment of *P. vivax* and *Plasmodium ovale* infections. Even today this recommendation is often not followed because there is a risk of severe haemolysis in persons with G6PD deficiency, and testing for G6PD deficiency is not generally available.

As malaria programmes progress towards the elimination of *Plasmodium falciparum* malaria, the proportion of malaria infections attributable to *P. vivax* outside of sub-Saharan Africa increases [3]; *P. vivax* is more difficult to eliminate because of relapse [4–6]. To eliminate *P. vivax,* relapses must be prevented with radical curative treatment regimens. The haemolytic effect of 8-aminoquinolines is dose dependent. High doses can cause significant haemolysis even in those with intermediate levels of G6PD deficiency [7]. With increased use of radical curative treatment, it is important to have a clear picture of haemolysis caused by 8-aminoquinolines not only in persons who are fully G6PD deficient (hemizygotes, homozygotes), but also in those with intermediate degrees of deficiency (heterozygotes).

## Historical aspects of G6PD deficiency

G6PD deficiency is today a textbook topic in human biochemical genetics and in pharmacogenetics; but long before these became academic subjects, manifestations of G6PD deficiency had been recognized clinically since antiquity. Apart from anecdotes revolving around the philosopher and mathematician Pythagoras (5th century B.C.), the occurrence of episodes of severe anaemia associated with jaundice and dark urine was reported in the 19th century in Portugal, Italy, and Greece [8]. These episodes were attributed correctly to ingestion of fava beans, hence the term *favism*. The same symptoms were also found to occur with use of pamaquine (plasmoquine), an 8-aminoquinoline developed by the Germans after World War I and used for the treatment of malaria [9, 10]. Pamaquine was not well tolerated prompting investigation for alternative safer treatments for military use during World War II. This resulted eventually in the development of primaquine (an analogue of pamaquine) in 1950 by the US-based malaria research programme [10, 11]. Primaquine was better tolerated than pamaquine but ‘primaquine sensitivity’ was observed in some patients who became anaemic and jaundiced when taking this medication. Investigations revealed that in both favism and ‘primaquine sensitivity’ there was an acute haemolytic anaemia (AHA) with high serum bilirubin and often haemoglobinuria (“blackwater”). At the time, there was no obvious link between the two syndromes.

The link became clear only after Carson and colleagues [12] reported that in the red blood cells of subjects with a documented history of ‘primaquine sensitivity’ the enzyme activity of the erythrocyte G6PD was markedly decreased compared to appropriate controls. This was a landmark discovery, as it identified for the first time a red blood cell enzymopathy with serious and distinctive clinical implications (some 20 more red cell enzymopathies have been discovered since) [13]. Very promptly Sansone and Segni [14] tested patients with a history of favism and found that they too had very low G6PD activity in their red blood cells. It was also observed that newborns with low levels of G6PD in their red blood cells had an increased frequency of neonatal jaundice, which was often severe [15]. Today, the term G6PD deficiency is appropriately used for this genetic trait, which carries the risk of severe neonatal jaundice and of AHA upon exposure to primaquine (as well as to some other drugs) or ingestion of fava beans.

## G6PD deficiency: from genotype to phenotype

**Fig. 1:**
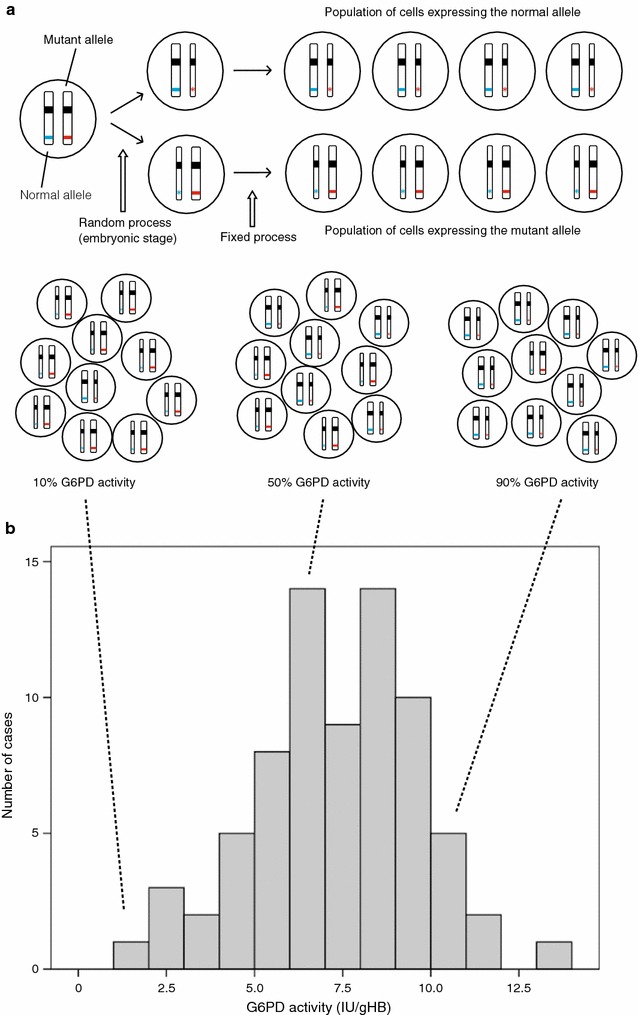
Somatic cell mosaicism in G6PD heterozygous females and the associated G6PD activity (phenotype). X-chromosome inactivation and the phenotypic expression of G6PD deficiency in heterozygotes for *GPPD* mutations (**a**) (was Adapted from Baird et al. [61]). The top panel shows that at an early stage during embryonic development in each somatic cell of a female one of the two X chromosomes is inactivated (symbolized by a thin chromosome). In a heterozygote with one normal *G6PD* allele (blue) and one mutant (deficient) *G6PD* allele (red), after X-chromosome inactivation there are two types of cells: one type (top), where only the normal allele is expressed (blue stripe) will be G6PD normal; the other type (bottom) where only the mutant allele is expressed (red star), will be G6PD deficient. Once X inactivation has taken place it is faithfully maintained in the progeny of each cell. The bottom panel illustrates that, because X inactivation in the embryo is a random process, in adult tissue (e.g. red blood cells) the ratio between the number of cells in which one X-chromosome is inactive to the number of cells in which the other X-chromosome is active is variable: in these examples 1:9 (left), 5:5 (middle), 9:1 (right) (**b**) (was adapted from Bancone et al. [62]). This figure illustrates the distribution of G6PD activity in red cells from 74 G6PD heterozygous females. The G6PD activity is highly variable. The median activity is 11.76 IU/gHb so that 12 females, though heterozygous, are in the normal range, i.e. they appear to be G6PD normal (*extreme phenotype*). On the other hand, five females have ≲30% of the median activity, i.e. they are almost as G6PD deficient as a hemizygous male (*extreme phenotype*). The remaining females have intermediate G6PD levels. The dotted lines linking Fig. 1a to b show graphically how the extreme and intermediate red cell phenotypes arise

Since the *G6PD* gene maps to the X chromosome (of which males have only one), a male with a mutation (called a *hemizygote*) causing G6PD deficiency will have full expression of the defect. In contrast, a female (having two X chromosomes), may have a normal *G6PD* gene on one chromosome and a mutated *G6PD* gene on the other chromosome, in which case she is called a *heterozygote*. If G6PD deficiency were autosomal (like most other enzymopathies) rather than X-linked, the heterozygous state probably would not matter very much, or at all. Indeed, having roughly 50% of normal enzyme in all cells is “good enough” with respect to most enzymes (Fig. 1a). For an X-linked gene, the situation is made radically different because of the phenomenon of random X-chromosome inactivation (*lyonization*) whereby, in each one of the somatic cells of a female, only the genes from one X chromosome are expressed, while those from the other are silenced. Thus, in some cells only the maternal X-linked genes will be expressed whereas in others only the paternal genes are expressed (a few genes do escape silencing, but *G6PD* is not one of them). Therefore, a female heterozygous for G6PD deficiency, rather than having about 50% G6PD activity in every red cell, has in her blood a mixture of G6PD normal and G6PD deficient red cells (Fig. 1a); this situation is referred to as *somatic cell mosaicism*.

To complicate matters further, the ratio of the two cell types that make up the mosaic is not the same in all females. X-inactivation takes place through a seemingly random process early in embryonic life, when there are few cells in the developing embryo. Therefore, although the ratio of the two cell types is normally distributed, the distribution is rather wide (Fig. 1b). This means that at the upper end of the distribution females have nearly all normal red blood cells whereas at the lower end they have nearly all G6PD deficient red blood cells: this state—called an extreme phenotype—mimics a deficient homozygote.

The clinical implications of these facts are important, to a large extent predictable, and validated by clinical experience. Since the average proportion of G6PD deficient red cells in heterozygotes is 50%, in the majority of these females AHA triggered by fava beans or by primaquine will be less severe than in hemizygous males. However, a female with an extreme (homozygous deficient-like) phenotype will develop AHA which is as severe as a hemizygous male (when receiving the same dose). Indeed, every major series of children with favism includes girls, the majority of them heterozygotes; some of whom had very severe favism requiring urgent blood transfusion. The published data on primaquine use in heterozygotes are scanty, because during its early evaluations primaquine was used by combat troops (then all male) and because males were selected during the early experimental work evaluating primaquine in G6PD deficient persons. However, in paediatric clinical trials of the anti-malarial chlorproguanil-dapsone (Lapdap^®^) carried out early in this century (the sulphone dapsone is potentially haemolytic in G6PD deficiency), AHA was seen in the majority of 200 heterozygous girls [16, 17]. As expected, the severity of AHA covered the full range from very mild to a severity similar to that seen in G6PD deficient (hemizygous) boys [18].

## G6PD deficiency: from phenotype to genotype

**Fig. 2:**
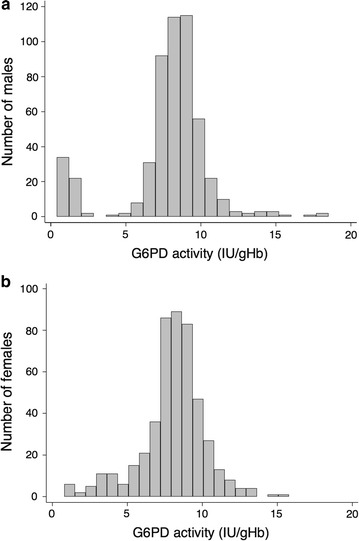
Phenotypic differences in quantitative G6PD activity between males and females. In a male population (**a**) there are two evident phenotypes (G6PD normal and deficient) as shown by the clearly bimodal distribution in the histogram. In a female population (**b**) some will have an intermediate phenotype as shown by the continuous distribution. (This figure was adapted from Oo et al. [26])

The erythrocytic G6PD activity decreases physiologically as erythrocytes age in the circulation. Therefore, what is normally measured in a blood sample haemolysate is the *average* activity, resulting from a mixture of younger red blood cells with higher activity and older cells with much less activity. The enzyme activity measured is often referred to as the G6PD phenotype (although more rigorous phenotypic features also include enzyme kinetic properties, pH-dependence, in vivo stability, thermostability, etc.); in G6PD normal subjects it is often around 7–10 IU/gHb (≳80% of normal, as determined by the population median). For example, if in a previously untested male G6PD activity of 1.8 IU/gHb (approximately ≲30% of normal) is measured, this is recognized as an obviously G6PD deficient phenotype. In a male population the two phenotypes (G6PD normal and G6PD deficient) are separated clearly (Fig. 2a). In a female population instead, whereas many will have a G6PD normal phenotype and very few (the square of the gene frequency in men) will have a fully G6PD deficient phenotype (as in males), quite a number (depending on the frequency of the G6PD deficiency gene(s) in that population) will have a phenotype which can aptly be called *intermediate* (≳30–≲80% of normal) (Fig. 2b). It is clear that males with a normal G6PD phenotype are hemizygous for the normal *G6PD* allele (genotype *G6PD*-*B*), whereas males with a G6PD deficient phenotype are hemizygous for a mutant allele (e.g*. G6PD*-*Mahidol,* or *G6PD*-*Med,* or *G6PD*-*A*-, depending on which allele is common in the respective population). This can be easily verified by molecular genotyping. Indeed, for certain studies this is an attractive approach, because samples can be conveniently batched and easily stored and no phenotypic quantitative assays are needed. However, there is an important proviso: either the entire *G6PD* coding region is sequenced, or there is reliable information on G6PD deficiency mutations present in the population. Failing that, because there are many different mutations associated with an abnormal phenotype, samples that are G6PD deficient might be mis-classified as normal if restricted genotyping is employed (and this has happened before).

The situation is rather different for females. Those with an intermediate phenotype will almost certainly be heterozygotes, but there will be significant overlap at both ends of the intermediate phenotype distribution. Some of those with a G6PD normal phenotype (≳80% of normal) might not be homozygous normal (*G6PD*-*B/G6PD*-*B* homozygotes) but heterozygotes (e.g. *G6PD*-*B/G6PD Mahidol*) with an ‘extreme phenotype’. For the same reason, some of those with a severely deficient phenotype (≲30% of normal) might also be heterozygotes, rather than homozygous for a *G6PD* deficient allele (e.g. *G6PD*-*Mahidol/G6PD*-*Mahidol*). Thus, the only way to identify heterozygosity definitively in all cases is by DNA analysis. However, this does not mean that DNA analysis is superior—rather the opposite. The heterozygous genotype is created by inheritance, whereas the phenotype is determined subsequently by the pattern of X chromosome inactivation. Importantly, it is the phenotype that matters from the clinical point of view. In other words, knowing that a female is heterozygous by genotype does not predict whether she may have severe haemolysis with primaquine, but knowing that she has a large proportion of G6PD deficient red blood cells (approximately 30% or less) will be predictive. That proportion can be assessed by qualitative or quantitative tests.

## Testing for G6PD deficiency

It is very important to be able to identify conveniently and accurately, at the point of care (i.e. in the clinic setting or during population surveys), subjects with an enzyme deficiency that would result in clinically significant AHA. Ideally the test must be easy to carry out and easy to interpret. Historically, the first simple phenotypic tests for detecting G6PD deficiency were the methaemoglobin reduction test (MRT) [19] and the dye decolourization test [20]. These were generally reliable and inexpensive. However, they required some basic laboratory equipment and took several hours to read. Subsequently, a fluorescent spot test (FST) was developed by Fairbanks and Beutler [21, 22]. With this test the naturally fluorescent NADPH produced by G6PD is detected under UV light; G6PD normal samples produce a bright green fluorescent spot whereas G6PD deficient samples will not. The test has shown remarkable stability in different settings. The FST has over 95% sensitivity and specificity in diagnosing any G6PD deficient sample with ≲30% normal activity [23–26]. With a simple two step procedure and a processing time of 30 min, the FST has become understandably one of the most widely used G6PD tests worldwide. For field use, the main limitations of the FST are that (i) a cold chain is required for receiving and storing reagents, (ii) electricity is required to light the UV lamp, and (iii) trained personnel are needed to interpret the result. In the past decade, two lateral flow rapid diagnostic tests (RDT) for G6PD have appeared on the market; the Binax Now™ and Carestart G6PD™. The Binax Now™ has over 97% sensitivity and specificity [27–29], but the operating temperature range (18–25 °C) is too narrow for use in tropical field settings. The Carestart G6PD™ (which is based on G6PD-mediated conversion of a soluble tetrazolium dye to a purple formazan precipitate) has been validated in different settings both in healthy volunteers and in malaria patients [23–26, 30–33]. Today, this is the only lateral flow RDT available for the phenotypic diagnosis of G6PD deficiency; shortcomings include no control line, as well as storage and operating temperatures which are not always within the temperature range prevailing in tropical regions.

Current G6PD phenotypic screening tests (relying on visual assessment) are not designed to detect heterozygous females with intermediate activity (approximately > 30% to approximately 80% of normal activity). The majority of these females are diagnosed as “G6PD normal” by both the FST and the CareStart™. A quantitative technique is required to detect the intermediate levels of G6PD activity and up to now this has been done for research purposes or as an advanced diagnostic methodology for rare cases. The gold standard for quantitative measurement of red cell G6PD enzymatic activity is the spectrophotometric assay [34] on blood haemolysates. In heterozygous females this will measure a weighted average of the activities of the two red blood cell populations (G6PD normal and G6PD deficient). The flow-cytometric read-out of the MRT [35] is a promising assay for the detection of G6PD activity at the single red blood cell level that assesses the actual proportion of G6PD normal and deficient red cell populations [36]. The spectrophotometric assay is quite straightforward, but it requires skilled laboratory technicians, specialized laboratory equipment, and reagents. Only very recently has a version of this assay been adapted to become a point of care (POC) quantitative test (Biosensor). This is currently under assessment in different settings [37, 38].

## AHA caused by 8-aminoquinolines and other agents

The earliest studies on primaquine dosing (which began in the early 1950s) showed that a 22.5 mg daily dose for 14 days was efficacious in preventing *P. vivax* relapse [11]. Subsequent studies, supported by extensive clinical use in soldiers with long latency Korean *P. vivax* infections, provided the rationale for a 15 mg daily dose for 14 days (0.25 mg/kg/day for 14 days; total dose 3.5 mg/kg) [39] in G6PD normal patients, which to this day is the most commonly used and frequently studied dosing regimen [40–43]. The lower efficacy of this regimen in the frequent relapse Chesson phenotype prevalent in East Asia and Oceania was soon recognized. More recently in this region, higher doses of primaquine (0.5 mg/kg/day for 14 days; total dose 7 mg/kg) have been recommended to prevent relapse. For identified G6PD deficient persons with ≲30% activity (with current phenotypic tests), the 14-day course of daily primaquine for radical cure is considered contraindicated. In these patients an alternative is to give primaquine 0.75 mg/kg/once weekly for 8 weeks (total dose 6 mg/kg). Evidence supporting this dose in G6PD deficient patients with the African A-variant was published over 50 years ago [44, 45]. The safety of the weekly dose in patients who are more severely G6PD deficient with other variants requires further verification [46, 47]. Despite the limitations, the weekly dose is widely recommended, although adherence to this recommendation varies.

Whilst the biochemical genetics of G6PD deficiency are well understood, data are scarce on the haemolytic effects of daily primaquine doses in G6PD heterozygous females with intermediate levels of G6PD activity (~ 30–80%), who would have a “normal” result using current G6PD deficiency rapid tests. In 1958, haemolysis during daily primaquine administration in G6PD heterozygous females of African descent (presumably with the African A-variant) was first reported. The proxy method used in lieu of a G6PD assay was the glutathione stability test. An important finding from this study was that among females with intermediate levels of stability some had developed haemolysis in vivo suggesting that “*cells of ‘intermediates’ that undergo hemolysis are fully as sensitive to primaquine as are the cells of ‘reactors’.* In 1962, it was confirmed that among females of African descent about 20% had haemolysis when given 30 mg of primaquine daily [48], but this was found to be highly variable: “*In some hemolysis was only detectable by isotopic labelling of the erythrocytes whereas, in others the hemolytic susceptibility and the biochemical abnormalities of the erythrocytes were as severe, possibly even more severe, than in males with full expression*.”

**Fig. 3:**
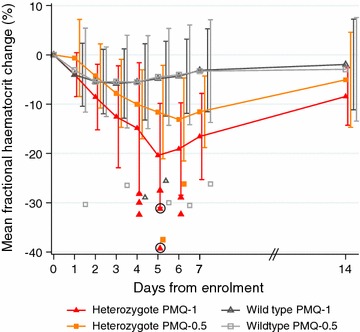
Mean fractional haematocrit changes over time in G6PD heterozygous and wild-type females taking primaquine. The line graph represents the fractional haematocrit plotted as the mean (95% CI). The plotted shapes represent individuals with maximum fractional haematocrit reductions below − 25%. The circled shapes represent individuals who received a blood transfusion. *Het* heterozygote, *WT* wild type, *PMQ-1* primaquine dosed at 1 mg/kg/day for 7 days, *PMQ-0.5* primaquine dosed at 0.5 mg/kg/day for 14 days. (This figure was taken from Chu et al. [49])

These astute observations by scientific pioneers are still relevant today. In a nested cohort study published earlier this year, dose-dependent haemolysis (independent of malaria associated haemolysis) was observed in G6PD Mahidol heterozygous females during primaquine administration for the radical curative treatment in acute *P. vivax* malaria [49] (confirming findings already observed half a century ago). Greater haematocrit reductions were observed in the patients taking 1 mg/kg/day for 7 days (the experimental alternative very high dose primaquine regimen) compared to 0.5 mg/kg/day for 14 days (the commonly used high dose primaquine regimen) (Fig. 3). Whilst the total dose was the same in the very high dose group, the daily dose was doubled. Two females in the very high dose primaquine group required blood transfusion; only one (with a pre-treatment haematocrit over 30%) complained of symptoms. There was a wide range of haemolysis resulting from primaquine in these G6PD Mahidol heterozygotes, as observed previously with dapsone in African A-heterozygotes [7]. Overall, haematocrit reductions were not associated with any specific clinical symptoms (other than those associated with anaemia itself). This has been found previously with primaquine [39], and more recently with dapsone [7, 50–52]. The largest study of drug-induced haemolysis in G6PD deficiency heterozygotes was following administration of dapsone rather than primaquine. In 200 heterozygotes for the G6PD A- variant the spectrum of haemolysis ranged from undetectable to quite severe, (see Fig. 6 in ref 7) as would be expected from inspection of Fig. 1b in this paper.

Tafenoquine, another 8-aminoquinoline derivative, was developed in the 1980’s as an alternative to primaquine [53, 54]. Its excellent efficacy against *P. vivax* has been established recently in large clinical trials [55]. Recently the effects of tafenoquine in G6PD deficient heterozygotes with G6PD activities in the range of 40–60% of normal were reported [56]. The extent of haemolysis was dose-dependent with greater haemoglobin reductions in patients receiving 300 mg compared to those receiving 200 mg or 100 mg. With 300 mg of tafenoquine, the haematologic changes were similar to those in heterozygote females who received primaquine 15 mg for 14 days (approximately 0.25 mg/kg/day). These heterozygotes did not develop clinical symptoms other than those associated with anaemia, and none of them required blood transfusion. Tafenoquine has a long terminal elimination half-life which allows a single dose to be given. Thus, unlike primaquine which can be stopped at the first signs of toxicity, tafenoquine cannot be stopped. One might expect that the haemolysis resulting from daily primaquine dosing will be mitigated by the fact that the oldest most vulnerable red blood cells are replaced by younger red blood cells that are less vulnerable because they have a higher G6PD activity. This should also be true for the longer acting tafenoquine. However, ‘self-limited haemolysis’ (when using daily primaquine) has been demonstrated in hemizygous males with the African G6PD A- variant, the Mahidol variant, and the Viangchan variants [44] and in heterozygous females with the G6PD Mahidol or G6PD Viangchan variants [49, 57] but not with more severe variants. In G6PD Mahidol heterozygotes the tafenoquine dose escalation study was stopped at 300 mg because of significant haemolysis.

Commonly prescribed drugs such as nitrofurantoin, quinolones (nalidixic acid and ciprofloxacin), rasburicase, and other agents (methylene blue) also are known to cause haemolysis in G6PD deficient persons, including heterozygotes [58, 59]. Infections, such as malaria, may also cause haemolysis, which can overlap with drug-induced AHA. Iatrogenically induced haemolysis in general is fully preventable and avoiding the causative drugs reduces unnecessary morbidity and mortality.

## Considerations for the use of 8-aminoquinolines in G6PD heterozygous females

Results from a number of G6PD deficiency tests give a binary “normal” or “deficient” result in female heterozygotes, when in fact, G6PD activity ranges on a continuous scale from severe deficiency to normal. In females any threshold between normal and deficient is arbitrary. Currently two arbitrary thresholds have been used: a) if the G6PD activity is ≲30% of normal females receive the same treatment as G6PD deficient males; b) if the G6PD activity is ≳70% of normal, females receive the same treatment as G6PD normal males. Among the million persons who have received primaquine in radical curative doses during mass drug administrations, only 16 persons (mostly likely G6PD deficient) were reported as having experienced severe haemolysis or anaemia [57]. In Latin America and the Caribbean (where the prevalence of G6PD deficiency is 4% and the most common variants are African A- and Mediterranean), no cases of primaquine associated haemolysis in females have been reported [60]. The low rates of severe AHA following primaquine may be in part explained by recognition of adverse effects (e.g. dark urine) and then stopping the medication. For heterozygous females with G6PD activity between ≳30 and ≲80% of normal (about one-half of all heterozygotes), there are no current evidence-based recommendations. There are very few data on the occurrence of mild to moderate anaemia in G6PD heterozygous females (see those described above). Symptoms may be missed or not reported unless haematologic parameters are measured before and after primaquine treatment. Thus, in known G6PD heterozygote females it would be reasonable to either withhold primaquine (or tafenoquine); or to accept that haemolysis will develop, monitor the individual carefully, and stop if adverse effects occur (keeping in mind that tafenoquine cannot be stopped).

## Conclusions: The future of primaquine and tafenoquine use

Over the past 90 years 8-aminoquinolines have been prescribed mostly without testing for G6PD deficiency: initially, because this enzyme defect was unknown; and subsequently because it was largely disregarded. Nowadays there is increasing deployment of semi-quantitative tests, which identify male hemizygotes and female homozygotes, but fail to identify a substantial proportion of female heterozygotes, some of whom have a risk of clinically significant iatrogenic haemolysis. At the moment, for most malaria-endemic areas where testing is unavailable and primaquine is the only option, radical treatment requires a careful appraisal of risks and benefits, consideration of safer treatment regimens (such as once weekly), and education of the patient to stop taking primaquine if adverse effects occur. This risk-benefit assessment requires knowledge of local relapse patterns and of G6PD variants and their severity, and of the availability of medical supervision and access to facilities for blood transfusion. In the future it is hoped that point of care quantitative tests will be developed and deployed. This will provide accurate assessment of the phenotype, and thus of the potential severity of haemolysis, which is roughly inversely proportional to G6PD activity: the lower the activity, the more severe the haemolysis. These tests will be especially necessary for the safe use of tafenoquine.

## Abbreviations

ACT: artemisinin-based combination therapy
AHA: acute haemolytic anaemia
FST: fluorescent spot test
G6PD: glucose-6-phosphate dehydrogenase
MRT: methaemoglobin reduction test
POC: point of care
RBC: red blood cell
RDT: rapid diagnostic test
